# WNT and TGF-Beta Pathway Alterations in Early-Onset Colorectal Cancer Among Hispanic/Latino Populations

**DOI:** 10.3390/cancers16233903

**Published:** 2024-11-21

**Authors:** Cecilia Monge, Brigette Waldrup, Francisco G. Carranza, Enrique Velazquez-Villarreal

**Affiliations:** 1 Center for Cancer Research, National Cancer Institute, Bethesda, MD 20892, USA; 2 Department of Integrative Translational Sciences, Beckman Research Institute of City of Hope, Duarte, CA 91010, USA; 3 City of Hope Comprehensive Cancer Center, Duarte, CA 91010, USA

**Keywords:** early-onset colorectal cancer, WNT pathway, TGF-beta pathway, Hispanic/Latino populations, ethnic disparities, genetic mutations, precision medicine

## Abstract

Background/Objectives: One of the fastest-growing minority groups in the U.S. is the Hispanic/Latino population. Recent studies have shown how this population is being disproportionately affected by early-onset colorectal cancer (CRC). Compared to corresponding non-Hispanic White (NHW) patients, Hispanic/Latino patients have both higher incidence of disease and rates of mortality. Two well-established drivers of early-onset CRC in the general population are alterations in the WNT and TGF-Beta signaling pathways; however, the specific roles of these pathways in Hispanics/Latinos are poorly understood. Methods: Here, we assessed CRC mutations in the WNT and TGF-Beta pathways by conducting a bioinformatics analysis using cBioPortal. Cases of CRC were stratified both by age and ethnicity: (1) early-onset was defined as <50 years vs. late-onset as ≥50 years; (2) we compared early-onset in Hispanics/Latinos to early-onset in NHWs. Results: No significant differences were evident when we compared early-onset and late-onset CRC cases within the Hispanic/Latino cohort. These results are consistent with findings from large cohorts that do not specify ethnicity. However, we found significant differences when we compared early-onset CRC in Hispanic/Latino patients to early-onset CRC in NHW patients: specifically, alterations in the gene bone morphogenetic protein-7 (BMP7) were more frequent in early-onset CRC for the Hispanic/Latino patients. In addition to these findings, we observed that both NHW patients and Hispanic/Latino patients with early-onset disease had better clinical outcomes when there was evidence of WNT pathway alterations. Conversely, the absence of TGF-Beta pathway alterations was uniquely associated with improved outcomes exclusively in early-onset Hispanic/Latino patients. Conclusions: In toto, these findings underscore how the WNT and TGF-Beta pathways may act differently in different ethnic groups with early-onset CRC. These findings may set a stage for developing new therapies tailored for reducing cancer health disparities.

## Simple Summary

Our study investigates how molecular changes in two important biological pathways, WNT and TGF-beta, differ between Hispanic/Latino and non-Hispanic White (NHW) patients with early-onset colorectal cancer. We found that certain genetic mutations in these pathways were more common in Hispanic/Latino patients, which may influence how the cancer develops and responds to treatment. By highlighting these differences, our research aims to improve understanding of why colorectal cancer appears to affect different ethnic groups in distinct ways. These findings could help guide the development of more personalized, effective treatments for Hispanic/Latino patients, an underrepresented group in cancer research.

## 1. Introduction

Colorectal cancer (CRC) is the third most prevalent cancer in the U.S. and the second leading cause of cancer-related death globally [1]. Although CRC has been historically associated with older age, recent data highlight a concerning rise in the incidence of CRC in younger individuals (<50 years) [2]—i.e., early-onset CRC. For example, individuals aged 18–40 and 40–50 are increasingly being diagnosed with rectal cancer [3]. Accordingly, other studies have shown that the incidence of early-onset CRC is rising at an annual rate of approximately 1.4% in individuals under 50 [4]. The rise in early-onset CRC has been observed both in the U.S. and globally [5]. However, the trend in early cases is especially pronounced in high-income countries, like the U.S.: early-onset CRC now represents approximately 10% of all new CRC cases [6,7]. In response, the American Cancer Society recently updated its screening guidelines and lowered the recommended starting age from 50 to 45 [4]. To compound the urgency of detecting early-onset CRC, the cancer itself is associated with more advanced stages at time of diagnosis and poorer histological differentiation. Both factors contribute to worse outcomes for younger patients [8,9]. These findings stress the importance of revising screening strategies towards early detection.

In addition to its alarming rise in the general population, early-onset CRC disproportionately affects one of the fastest growing minority groups in the U.S.—the Hispanic/Latino population [10,11]. This population often faces disparities in cancer outcomes and healthcare access [12,13]. The exact cause of the disparity in early-onset CRC outcomes is not only poorly understood but also involves a complex interplay of multiple factors. For instance, the role of unhealthy dietary patterns, such as the high consumption of processed foods, can accelerate the development of precancerous colorectal polyps [14]. In addition, genetic predispositions are significant contributors. Ionescu [15] points out that early-onset CRC (vs. late-onset) often presents with distinct oncogenic mutations and histopathological characteristics. Importantly, only a handful of studies have comprehensively characterized the Hispanic/Latino population in terms of the molecular drivers of CRC. This population is notably underrepresented in publicly available genomic databases [16].

**Figure 1:**
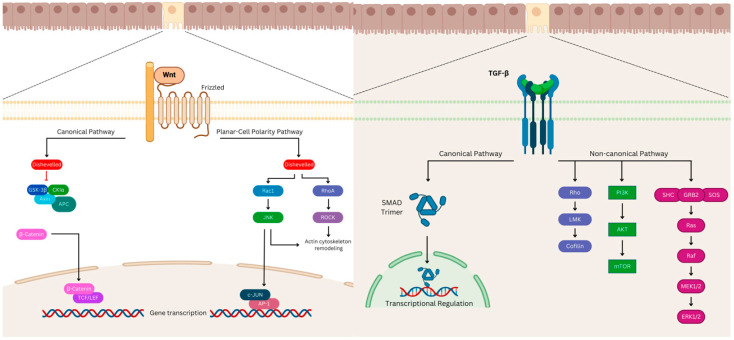
Illustration of the WNT signaling pathway on the left and the TGF-beta signaling pathway on the right.

Two signaling pathways are well known for harboring molecular targets implicated in CRC: the WNT signaling pathway and the TGF-beta signaling pathway (Figure 1).

While both pathways are recognized drivers of CRC progression, the molecular characteristics of early-onset CRC in Hispanic/Latino populations have not been defined sufficiently. Prior research has identified unique molecular traits in young-onset CRC: CIMP-low and LINE-1 hypomethylation [17]. Yet only a handful of reports have documented pathway-specific alterations in Hispanic/Latino patients. In this study, we aim to define the prevalence of WNT and TGF-beta pathway alterations in early-onset CRC among Hispanic/Latino patients, comparing these findings with both late-onset Hispanic/Latino patients and early-onset NHW patients. By leveraging bioinformatics analyses of publicly available CRC datasets, we seek to identify ethnic-specific molecular alterations in these key pathways, shed light on potential drivers of CRC in Hispanic/Latino patients, and pave the way for developing new treatment strategies for early-onset CRC in underrepresented populations.

## 2. Materials and Methods

We assessed individual patient-level clinical and genomic data from 19 colorectal cancer (CRC) datasets available in the cBioPortal database. We included studies categorized as colorectal adenocarcinoma, colon adenocarcinoma, and rectal adenocarcinoma, along with the GENIE BPC CRC v2.0-public dataset. We excluded two studies that focused on metastatic colorectal cancer samples. After selecting the relevant studies, we filtered the samples using the following five criteria. (1) Ethnicities were filtered to include Hispanic or Latino, Spanish, NOS; Hispanic, NOS; Latino, NOS; Mexican or Spanish surname only. (2) Sample we assessed were primary tumors. (3) Cancer type was filtered to include colon adenocarcinoma, rectal adenocarcinoma, and colorectal adenocarcinoma. (4) Histology was adenocarcinoma, NOS. (5) We included only one sample per patient. This filtering process yielded three datasets that met all criteria: TCGA PanCancer Atlas, MSK Nat Commun 2022, and GENIE BPC CRC. They comprised a total of 20 early-onset and 13 late-onset CRC samples from Hispanic/Latino patients. Additionally, data regarding age at diagnosis were retrieved from each patient’s individual clinical records within the GENIE database. Alterations in the WNT and TGF-beta pathways were defined as in a previous study [18].

Cohorts were defined based on age categories: Early-onset patients were less than 50 years old and late-onset patients were 50 years or older. Ethnicity was considered by classifying participants into two cohorts: Hispanics/Latinos and Non-Hispanic Whites (NHWs). Within these broader categories, participants were further stratified based on the presence or absence of WNT and TGF-beta pathway alterations. This allowed us to perform a more granular analysis of the impact of these genetic factors on the cohorts. To assess differences between these defined cohorts, Chi-square tests were employed to evaluate the independence of categorical variables. This statistical approach allowed us to examine the association between age, ethnicity, and the presence of pathway alterations. Additionally, further sub-stratification was conducted based on tumor location; we distinguished colon from rectal cancers. This detailed stratification allowed us to comprehensively analyze how age, ethnicity, and tumor location interact with molecular alterations. It thus provided insights into the potential heterogeneity in patient outcomes and treatment responses within the study population. Kaplan–Meier survival curves were utilized to evaluate overall survival by assessing the influence of WNT and TGF-beta pathway alterations. This method involved estimating the survival function from time-to-event data, allowing for the visualization of survival probabilities over time. Specifically, patients were stratified based on the presence or absence of WNT and TGF-beta alterations. The resulting survival curves were plotted, and the log-rank test was employed to compare differences between cohorts, determining statistical significance. Additionally, median survival times were calculated, and 95% confidence intervals were provided to quantify the uncertainty around these estimates. This comprehensive approach enabled a clear understanding of how these molecular alterations impact patient outcomes.

## 3. Results

The Hispanic/Latino cohort comprised 33 samples from three studies; while 36% of these patients were diagnosed at age 50 or older, 64% of them presented with early-onset CRC (Table 1). Colon adenocarcinoma accounted for 72% of cases, rectal adenocarcinoma for 21.2%, and colorectal adenocarcinoma for 6.1%. The cohort was predominantly male (60.6%). All samples analyzed were primary tumors to focus on the initial presentation of the cancer. At the time of diagnosis, 15.2% of patients were Stage II, 30.3% Stage III, and 39.4% Stage IV. A total of 69.7% of patients identified as Mexican (including Chicano); 15.2% were categorized as Hispanic or Latino; 9.1% were identified by a Spanish surname only; and 6.1% classified as Spanish NOS, Hispanic NOS, or Latino NOS.

In comparison, the NHW cohort consisted of 342 samples from three studies, primarily comprising late-onset patients; 80.7% were aged 50 and older (Table 2). Colon adenocarcinoma was again the most common diagnosis, representing 68.7% of cases, followed by rectal adenocarcinoma (28.9%) and colorectal adenocarcinoma (2.3%). Gender distribution in this cohort showed 53.5% males. All samples were primary tumors, emphasizing initial disease presentation. The stage at diagnosis revealed that 0.6% were Stage 0 (CIS), 2% Stage I, 1.8% Stage II, 6.4% Stage III, and 4.1% Stage IV. This comprehensive demographic analysis highlights significant differences in age, cancer type, and ethnic background between the early-onset Hispanic/Latino population and the NHW cohort, underscoring important trends in colorectal cancer presentation across these populations.

In our Hispanic/Latino cohort when comparing early-onset with late onset, the patients with early-onset CRC were, similarly, predominantly male compared to patients over 50 years of age (57.1 vs. 66.7%, *p* = 1). The patients with early-onset CRC were, similarly, frequently female compared to patients over 50 years of age (42.8% vs. 33.3%, *p* = 0.9). The median tumor mutational burden (TMB) was significantly higher in patients with early-onset CRC (median: 4.87, IQR: 1.89–21.38) compared to patients > 50 years of age (median: 3.8, IQR: 3.22–4.82) (*p* = 1). Patients with young-onset CRC (≤50 years old) had lower rates of TP53 (71.4% versus 75%, *p* = 1), APC (66.7 versus 91.7%, *p* = 0.2), and KRAS (38.1% versus 50%, *p* < 0.7) compared to patients over 50 years of age, but this was not statistically significant (Table 3).

In our early-onset Hispanic/Latino cohort when comparing with the Non-Hispanic White Cohort, the Hispanic/Latino patients with early-onset CRC were less frequently male compared to early-onset NHW (57.1 vs. 50.7%, *p* = 1). The patients with early-onset CRC were, similarly, frequently female compared to early-onset NHW patients (42.9% vs. 43.5%, *p* = 0.9). The median tumor mutational burden (TMB) was significantly higher in patients with early-onset Hispanic/Latino CRC patients (median: 4.87, IQR: 1.89–21.38) compared to early-onset NHW patients (median: 4.32, IQR: 2.53–7.56) (*p* = 1). Hispanics/Latinos patients with early-onset CRC had higher rates of TP53 (71.4% versus 68.1%, *p* = 1), APC (66.7 versus 52.2%, *p* = 0.5), and KRAS (38.1% versus 37.7%, *p* < 0.7) compared to early onset NHW patients, but this was not statistically significant (Table 4).

**Figure 2:**
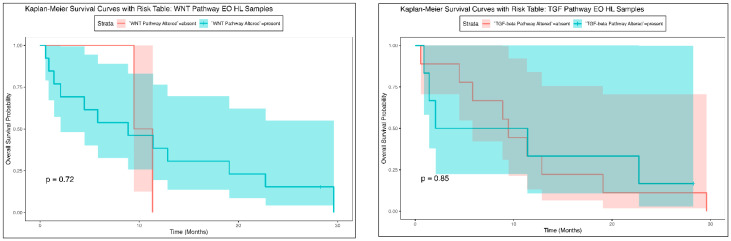
Overall survival curves of early-onset Hispanic/Latino patients stratified by the presence or absence of WNT (**left**) and TGF-β (**right**) pathway alterations.

The Kaplan–Meier survival analysis for Hispanic/Latino early-onset CRC patients showed no significant difference in overall survival between those with and without WNT pathway alterations (Figure 2). Both groups exhibited a similar decline in survival probability over time, with overlapping confidence intervals. Although patients with WNT pathway alterations experienced a more gradual decline in survival probability compared to those without alterations, who had a sharper decrease within the first 10 months, the survival curves converged by the end of the observation period. The *p*-value of 0.72 indicated that the difference in survival outcomes was not statistically significant, suggesting that WNT pathway alterations do not substantially impact overall survival in this cohort of early-onset Hispanic/Latino CRC patients. Similarly, Kaplan–Meier survival analysis comparing overall survival in early-onset Hispanic/Latino CRC patients with and without TGF-beta pathway alterations revealed no significant difference between the groups (Figure 2). The survival curves followed a nearly identical trajectory, with overlapping confidence intervals that indicated comparable survival probabilities over time. Patients with TGF-beta pathway alterations showed a slightly slower decline in survival probability early in the observation period, but the curves converged by the end. The *p*-value of 0.85 confirmed that the difference in survival between patients with and without TGF-beta pathway alterations was not statistically significant and these results suggest that alterations in the TGF-beta pathway do not significantly influence overall survival in this cohort of early-onset Hispanic/Latino CRC patients. Similar to NHW overall survival results (Figure S1), these findings highlight that, in this specific ethnic cohort, neither WNT nor TGF-beta pathway alterations are major determinants of survival outcomes in early-onset colorectal cancer.

In our Hispanic/Latino cohort, no significant difference was observed in the frequency of WNT pathway alterations between early-onset and late-onset CRC patients (90.5% vs. 91.7%, *p* = 1) (Table 3). This finding suggests that WNT pathway alterations are consistently prevalent across both age groups, potentially indicating a fundamental role for this pathway in the tumorigenesis of CRC within the Hispanic/Latino population, irrespective of age at onset. However, the rate of APC alterations was lower in early-onset CRC compared to late-onset patients, though this difference was not statistically significant (66.7% versus 91.7%, *p* = 0.2) (Table S1, Figure S2). Notably, several WNT pathway-related genes, including AXIN1, AXIN2, and RNF43, exhibited alterations in early-onset patients but no alterations in late-onset CRC patients. GSK3B was the only WNT pathway-related gene that showed no alterations in either age group. When stratified by cancer type (e.g., colon versus rectum adenocarcinoma), no significant differences in WNT pathway alterations were observed, indicating that these genetic variations are consistent across CRC subtypes within this ethnic cohort (Table S2). Similarly, TGF-Beta pathway alterations were not significant between early-onset and late-onset patients in the Hispanic/Latino cohort, nor did stratification by cancer type reveal any substantial variation in these pathway alterations. This suggests that, while some WNT pathway genes are more commonly altered in early-onset cases, these differences are largely independent of the specific CRC subtype.

When comparing early-onset Hispanic/Latino patients with early-onset NHW patients, significant differences were observed in WNT pathway alterations, including gene mutations within this well-established pathway associated with CRC (90.5% vs. 67.7%, *p* < 0.05) (Table 3). This finding suggests a potentially important role for the WNT pathway in the tumorigenesis of early-onset CRC within the Hispanic/Latino population. The frequency of APC alterations was lower in early-onset Hispanic/Latino patients compared to NHW patients, though this difference was not statistically significant (66.7% vs. 55.4%, *p* = 0.5) (Table S3, Figure S3). Alterations in AXIN1 and AXIN2 were similar between the two ethnic groups, while mutations in RNF43 were more frequent in early-onset Hispanic/Latino patients than in their NHW counterparts (23.8% vs. 7.7%, *p* = 0.1). When stratified by cancer type, early-onset Hispanic/Latino patients with colon adenocarcinoma showed a trend toward marginal significance in WNT pathway alterations compared to early-onset NHW patients (93.3% vs. 69%, *p* = 0.08), suggesting potential ethnic-specific variations in this pathway for colon cancer (Table S4). Although no significant differences were observed in overall TGF-Beta pathway alterations, a notable disparity in BMP7 mutations was identified: BMP7 mutations were absent in early-onset Hispanic/Latino CRC patients, whereas 18.5% of early-onset NHW patients exhibited these alterations (*p* < 0.03). BMP7, a key member of the bone morphogenetic protein family, plays a critical role in regulating cell growth and differentiation and has been implicated in CRC progression. The absence of BMP7 mutations in early-onset Hispanic/Latino patients suggests a distinct molecular profile that may influence tumor behavior or response to treatment in this population. These findings highlight key ethnic disparities in molecular pathways, particularly within the WNT and TGF-Beta pathways, associated with early-onset colorectal cancer.

## 4. Discussion

The WNT signaling pathway is essential for regulating stem cell renewal and cell fate in the intestinal epithelium [19,20,21,22]. WNT signaling is frequently dysregulated in CRC due to mutations in tumor suppressor genes: three examples are APC, AXIN1, and AXIN2. Such mutations lead to sustained WNT signaling, may grant self-renewing properties to cells, and can ultimately promote tumor progression [19,23,24]. In studies across broad patient populations, WNT signaling was a key driver of colonic carcinogenesis [19,25,26]. Additionally, WNT signaling appears to be a promising target for reducing cancer stem cell characteristics [27]. Such cells are considered an important subpopulation of cancer cells to target via new therapies, because they are resistant to traditional chemotherapy and may be responsible for relapse.

The TGF-beta signaling pathway similarly plays a crucial role in regulating cell proliferation and apoptosis in normal intestinal tissues. Under normal physiological conditions, TGF-beta functions as a tumor suppressor by inducing cell cycle arrest and promoting programmed cell death [28,29]. Conversely, aberrantly elevated TGF-beta levels have been strongly linked to both progression and poor clinical outcomes in CRC [30,31]. High TGF-beta expression has been correlated with advanced disease stages, greater recurrence risk, and poorer clinical outcomes [32,33,34]. Mechanistically, the progression of CRC is exacerbated by mutations in TGF-beta pathway genes, like BMP7; this mutation allows tumor cells to escape growth-inhibitory effects and evade apoptosis [35,36,37,38]. Taken altogether, both WNT and TGF-beta play well-documented roles in CRC for the general population of patients. Nevertheless, merely a handful of studies have probed how these pathways operate in Hispanic/Latino populations with early-onset CRC.

In our studies, ethnicity in early-onset CRC appeared to have an impact on WNT signaling. For example, our Hispanic/Latino patients (vs. NHW) had marginally higher rates of WNT pathway alterations. This was especially true in early-onset colon cancer (vs. rectal), which suggests a potential interaction between tumor site and ethnicity in CRC pathogenesis. Other, specific trends we observed in WNT pathway mutations in Hispanic/Latino patients include alterations in AXIN1, AXIN2, and RNF43. Interestingly, our study revealed no significant differences in the prevalence of APC, TP53, or KRAS mutations between early-onset Hispanic/Latino and NHW patients. These results are consistent with previous studies that suggest a lower frequency of APC mutations in younger CRC patients, particularly in early-onset CRC cases [17]. While our study provides valuable insights into the impact of ethnicity on molecular characteristics of early-onset CRC, it has several limitations which are important to note: the retrospective nature of the bioinformatics analysis, combined with potential selection bias from publicly available genomic databases, may limit the generalizability of our findings. Furthermore, the underrepresentation of Hispanics/Latinos in these databases [13,39] hinders our ability to draw comprehensive conclusions regarding molecular disparities in CRC across all Hispanic/Latino subpopulations. Larger prospective studies are needed to validate our findings and explore the underlying biological mechanisms driving these ethnic differences in CRC. Nevertheless, our results appear to align with emerging studies in this arena.

Recent studies have revealed other types of significant variations in molecular patterns among different ethnic groups. For instance, Pérez-Mayoral et al. [40] identified molecular and sociodemographic disparities in Hispanic/Latino populations, particularly in Puerto Rico, where genetic ancestry was shown to be a key factor in CRC development. Their findings indicated that individuals with higher levels of African ancestry had a greater likelihood of developing rectal tumors. Similarly, Yamada et al. [41] reported notable differences in differentially expressed genes (DEGs) and pathway utilization among White Americans, Alabama African Americans, and Oklahoma American Indians. These results suggest that distinct biological patterns in CRC may arise from ethnic and racial differences shaped by factors like diet, geography, and cultural practices. Therefore, ethnic diversity is crucial in CRC research in order to develop personalized strategies for prevention, diagnosis, and treatment.

In our studies, the frequency of BMP7 mutations was notably higher in early-onset Hispanic/Latino patients. Alterations in BMP7, a gene within the TGF-beta signaling pathway, were significantly more frequent in early-onset Hispanic/Latino patients (vs. NHWs). Accordingly, in prior studies, TGF-beta pathway dysregulation was associated with poorer clinical outcomes in CRC [32,34]. Interestingly, our data also suggest that the absence of TGF-beta pathway alterations in early-onset Hispanic/Latino patients is linked to improved survival; contrastingly, NHW patients with TGF-beta alterations are more strongly associated with aggressive disease. This difference may reflect ethnic-specific tumor biology and highlight the importance of tailored therapeutic strategies for Hispanic/Latino populations. The elevated TMB observed in early-onset Hispanic/Latino patients compared to late-onset patients suggests increased genomic instability in younger individuals, which may contribute to the higher incidence and poorer prognosis in this population. High TMB has been associated with better responses to immunotherapy, and these findings may have clinical implications for the development of targeted therapies in Hispanic/Latino CRC patients [11].

One limitation of our study is the relatively small sample size of early-onset Hispanic/Latino CRC patients compared to NHW patients. This reflects the broader challenge of limited genomic data availability for underrepresented populations, such as Hispanics/Latinos, in publicly accessible databases. Despite this constraint, our study provides critical insights into molecular disparities by leveraging one of the few available genomic datasets suitable for ethnicity-focused analyses. The significant differences observed, such as the higher frequency of WNT pathway alterations in early-onset Hispanic/Latino patients (90.5% vs. 67.7%, *p* < 0.05) and the absence of BMP7 mutations in this cohort compared to 18.5% in NHW patients (*p* < 0.03), underscore the distinct tumor biology within this population. These findings not only contribute to our understanding of the ethnic-specific molecular mechanisms driving early-onset CRC but also highlight the urgent need for larger, more diverse datasets to validate and expand upon these results. In addition, while our analysis included CRC cases spanning stages, all samples were primary tumors, ensuring a focus on the initial tumor biology; however, we acknowledge that differences in cancer stage may influence tumor mutational burden and molecular alterations, underscoring the need for future studies with larger sample size stratified by stage to validate and refine our findings. Addressing these gaps will be essential for developing precision medicine approaches tailored to the unique needs of underrepresented populations and for reducing cancer health disparities.

Our study focused exclusively on sporadic CRC cases as identified within the selected projects in the cBioPortal database, which do not provide sufficient clinical information to distinguish or include inflammatory bowel disease (IBD)-associated CRC. Consequently, our analysis was limited to sporadic CRC, precluding the exploration of potential etio-pathophysiological differences between these two distinct CRC types. Despite this limitation, our findings revealed significant disparities in molecular alterations between Hispanic/Latino and NHW patients with early-onset CRC. Future studies incorporating datasets that differentiate between sporadic and IBD-associated CRC are essential to fully elucidate the roles of WNT and TGF-Beta pathways in these distinct CRC etiologies and to further refine therapeutic strategies tailored to specific patient groups.

This study presents an in-depth analysis of the WNT and TGF-Beta pathways, critical drivers of CRC pathogenesis, to uncover ethnic-specific differences in early-onset CRC among Hispanic/Latino patients. While our focus was intentionally narrow in order to derive meaningful insights, this approach underscores the need for future studies to explore additional pathways and molecular targets in CRC disparities. This work provides a foundation for expanding bioinformatics research to enhance our understanding of CRC in diverse populations.

## 5. Conclusions

Our study highlights significant ethnic disparities in WNT and TGF-beta pathway alterations in early-onset CRC between Hispanic/Latino and NHW populations. The higher prevalence of WNT pathway alterations and TGF-B-pathway related gene BMP7 mutations in early-onset Hispanic/Latino CRC patients underscores the need for further investigation into the unique molecular drivers of CRC in this underserved population. As precision medicine continues to evolve, it is essential to address these disparities to develop more effective, personalized treatment strategies that improve clinical outcomes for Hispanic/Latino CRC patients.

## Figures and Tables

**Table 1:** Patient Demographics and Clinical Characteristics of the Hispanic/Latino (H/L) and Non-Hispanic White (NHW) cohorts.

Clinical Feature	H/L Cohort *n* (%)	NHW Cohort *n* (%)
Age *		
<50	21 (63.6%)	65 (19.0%)
≥50	12 (36.4%)	276 (80.7%)
Cancer Type		
Colon Adenocarcinoma	24 (72.7%)	235 (68.7%)
Rectal Adenocarcinoma	7 (21.2%)	99 (28.9%)
Colorectal Adenocarcinoma	2 (6.1%)	8 (2.3%)
Sex		
Male	20 (60.6%)	183 (53.5%)
Female	13 (39.4%)	159 (46.5%)
Sample Type		
Primary Tumor	33 (100.0%)	342 (100.0%)
Stage at Diagnosis **		
Stage 0 (CIS)	0 (0.0%)	2 (0.6%)
Stage I	0 (0.0%)	7 (2.0%)
Stage II	5 (15.2%)	6 (1.8%)
Stage III	10 (30.3%)	22 (6.4%)
Stage IV	13 (39.4%)	14 (4.1%)
Ethnicity		
Hispanic Or Latino	5 (15.2%)	0 (0.0%)
Mexican (includes Chicano)	23 (69.7%)	0 (0.0%)
Spanish NOS; Hispanic NOS, Latino NOS	2 (6.1%)	0 (0.0%)
Spanish surname only	3 (9.1%)	0 (0.0%)
Non-Spanish; Non-Hispanic	0 (0.0%)	342 (100.0%)

* NHW NA:1—Data for one Non-Hispanic White (NHW) individual was not available for this variable. ** HL NA: 5, NHW NA: 291—Data for this variable was missing in five Hispanic/Latino (HL) individuals. For 291 Non-Hispanic White (NHW) individuals, data for this variable was also unavailable.

**Table 2:** Age-related variations in clinical features in the Hispanic/Latino cohort. Ethnicity-associated differences in clinical features between early-onset Hispanic and Latino and Non-Hispanic White (NHW) cohorts.

Clinical Feature	Early-Onset H/L *n* (%)	Late-Onset H/L *n* (%)	*p*-Value	Early-Onset H/L *n* (%)	Early-Onset NHW *n* (%)	*p*-Value
Median Age (IQR)	41 (36–45)	63 (58–74)	2.56 × 10^6^	41 (36–45)	43 (38–47)	0.2149
Median mutation count	9 (6–10)	24 (7–63)	0.4414	9 (6–10)	58 (7–79)	0.01093
Median TMB (IQR) *	4.87 (1.89–21.38)	3.8 (3.22–4.82)	1	4.87 (1.89–21.38)	4.32 (2.53–7.56)	0.9215
Median FGA **	0.06 (0.04–0.28)	0.31 (0.10–0.36)	0.184	0.06 (0.04–0.28)	0.17 (0.06–0.34)	0.2275
Oncotree Code						
COAD	14 (66.7%)	10 (83.3%)	0.56	14 (66.7%)	38 (55.1%)	0.22336
COADREAD	2 (9.5%)	0 (0.0%)		2 (9.5%)	5 (7.2%)	
READ	5 (23.8%)	2 (16.7%)		5 (23.8%)	22 (31.9%)	
Sex						
Male	12 (57.1%)	8 (66.7%)	0.7188	12 (57.1%)	35 (50.7%)	0.9906
Female	9 (42.9%)	4 (33.3%)		9 (42.9%)	30 (43.5%)	
APC Mutation						
Present	14 (66.7%)	11 (91.7%)	0.2062	14 (66.7%)	36 (52.2%)	0.5114
Absent	7 (33.3%)	1 (8.3%)		7 (33.3%)	29 (42.0%)	
KRAS Mutation						
Present	8 (38.1%)	6 (50.0%)	0.7157	8 (38.1%)	26 (37.7%)	1
Absent	13 (61.9%)	6 (50.0%)		13 (61.9%)	39 (56.5%)	
TP53 Mutation						
Present	15 (71.4%)	9 (75.0%)	1	15 (71.4%)	47 (68.1%)	1
Absent	6 (28.6%)	3 (25.0%)		6 (28.6%)	18 (26.1%)	
BMP7 Mutation						
Present	0 (0.0%)	0 (0.0%)	1	0 (1200.0%)	12 (18.5%)	0.03361
Absent	21 (100.0%)	12 (100.0%)		1 (5300.0%)	53 (81.5%)	

* EO H/L NA: 17, LO H/L NA: 9, EO NHW NA: 42; ** EO H/L NA: 4, EO NHW NA: 1.

**Table 3:** Rates of WNT and TGF-Beta pathway alterations among early-onset and late-onset Hispanic/Latino CRC patients.

	Early-Onset H/L *n* (%)	Late-Onset H/L *n* (%)	*p*-Value
WNT Alterations Present	19 (90.5%)	11 (91.7%)	1
WNT Alterations Absent	2 (9.5%)	1 (8.3%)	
TGF Alterations Present	8 (38.1%)	4 (33.3%)	1
TGF Alterations Absent	13 (61.9%)	8 (66.7%)	

**Table 4:** Rates of WNT and TGF-Beta pathway alterations among early-onset Hispanic/Latino and Non-Hispanic White (NHW) CRC Patients.

	Early-Onset H/L *n* (%)	Early-Onset NHW *n* (%)	*p*-Value
WNT Alterations Present	19 (90.5%)	44 (67.7%)	0.04876
WNT Alterations Absent	2 (9.5%)	21 (32.3%)	
TGF Alterations Present	8 (38.1%)	17 (26.2%)	0.4071
TGF Alterations Absent	13 (61.9%)	48 (73.8%)	

## Data Availability

All data used in the present study is publicly available at https://www.cbioportal.org/ (accessed on 8 August 2024) and https://genie.cbioportal.org (accessed on 8 August 2024). Additional data can be provided upon reasonable request to the authors.

## Supplementary Materials

The following supporting information can be downloaded at: https://www.mdpi.com/article/10.3390/cancers16233903/s1, Figure S1. Overall survival curves of early-onset Non-Hispanic White (NHW) patients stratified by the presence or absence of WNT (left) and TGF-β (right) pathway alterations; Table S1. Alteration Rates of WNT and TGF-Beta Pathway-Related Genes Among Early-Onset and Late-Onset Hispanic/Latino CRC Patients; Figure S2. Counts of WNT and TGF-Beta Pathway-Related Gene Alterations in Early-Onset and Late-Onset Hispanic/Latino CRC Patients; Table S2. Rates of WNT and TGF-Beta pathway alterations in early-onset and late-onset Hispanic/Latino CRC patients, stratified by colon and rectal adenocarcinomas; Table S3. Alteration Rates of WNT and TGF-Beta Pathway-Related Genes in Early-Onset Hispanic/Latino and Non-Hispanic White CRC Patients; Figure S3. Counts of WNT and TGF-Beta Pathway-Related Gene Alterations in Early-Onset Hispanic/Latino and Non-Hispanic White CRC Patients. Table S4. Rates of WNT and TGF-Beta pathway alterations in early-onset Hispanic/Latino and Non-Hispanic White (NHW) CRC patients, stratified by colon and rectal adenocarcinomas.
