# Supplementary material for: WNT and TGF-Beta Pathway Alterations in Early-Onset Colorectal Cancer Among Hispanic/Latino Populations
Source: Cancers (Basel). 2024 Nov 21;16(23):3903. doi: 10.3390/cancers16233903 (PMC11639970; doi:10.3390/cancers16233903)
Supplement: Supplementary file 1 [file cancers-16-03903-s001.zip › cancers-3296613-supplementary.pdf]

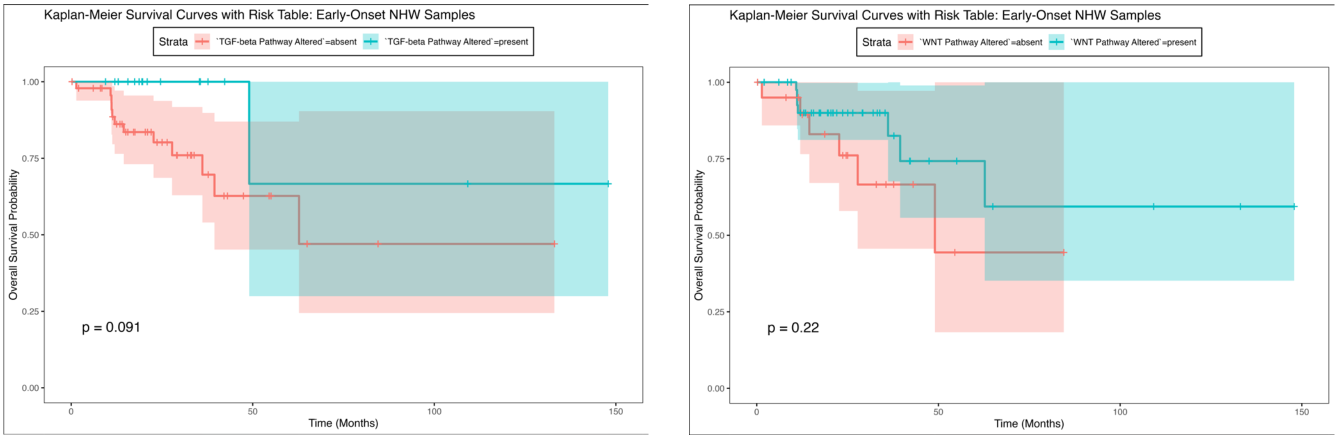

Figure S1. Overall survival curves of early-onset Non-Hispanic White (NHW) patients stratified by the presence or absence of WNT (left) and TGF-β (right) pathway alterations.

Table S1. Alteration Rates of WNT and TGF-Beta Pathway-Related Genes Among Early-Onset and Late-Onset Hispanic/Latino CRC Patients.

Gene	Early Onset H/L n (%)	Late Onset H/L n (%)	p-value
**APC Mutation**			
Present	14 (66.7%)	11 (91.7%)	0.2062
Absent	7 (33.3%)	1 (8.3%)	
**AXIN1 Mutation**			
Present	1 (4.8%)	0 (0.0%)	1
Absent	20 (95.2%)	12 (100.0%)	
**AXIN2 Mutation**			
Present	1 (4.8%)	0 (0.0%)	1
Absent	20 (95.2%)	12 (100.0%)	
**GSK3B Mutation**			
Present	0 (0.0%)	0 (0.0%)	1
Absent	21 (100.0%)	12 (100.0%)	
**RNF43 Mutation**			
Present	5 (23.8%)	0 (0.0%)	0.1329
Absent	16 (76.2%)	12 (100.0%)	
**TGFBR2 Mutation**			
Present	1 (4.8%)	0 (0.0%)	1
Absent	20 (95.2%)	12 (100.0%)	
**TGFB2 Mutation**			
Present	0 (0.0%)	0 (0.0%)	1
Absent	21 (100.0%)	12 (100.0%)	
**TGFBRAP1 Mutation**			
Present	0 (0.0%)	0 (0.0%)	1
Absent	21 (100.0%)	12 (100.0%)	
**TGFBR1 Mutation**			
Present	1 (4.8%)	1 (8.3%)	1
Absent	20 (95.2%)	11 (91.7%)	
**TGFBR3 Mutation**			
Present	0 (0.0%)	0 (0.0%)	1
Absent	21 (100.0%)	12 (100.0%)	
**TGFB1I1 Mutation**			
Present	0 (0.0%)	0 (0.0%)	1
Absent	21 (100.0%)	12 (100.0%)	
**TGFBI Mutation**			
Present	0 (0.0%)	0 (0.0%)	1
Absent	21 (100.0%)	12 (100.0%)	
**TGFB1 Mutation**			
Present	0 (0.0%)	0 (0.0%)	1
Absent	21 (100.0%)	12 (100.0%)	
**TGFBR3L Mutation**			
Present	0 (0.0%)	0 (0.0%)	1
Absent	21 (100.0%)	12 (100.0%)	
**TGFB3 Mutation**			
Present	0 (0.0%)	0 (0.0%)	1
Absent	21 (100.0%)	12 (100.0%)	
**TGFA Mutation**			
Present	0 (0.0%)	0 (0.0%)	1
Absent	21 (100.0%)	12 (100.0%)	
**SMAD1 Mutation**			
Present	0 (0.0%)	0 (0.0%)	1
Absent	21 (100.0%)	12 (100.0%)	
**SMAD2 Mutation**			
Present	1 (4.8%)	0 (0.0%)	1
Absent	20 (95.2%)	12 (100.0%)	
**SMAD3 Mutation**			
Present	0 (0.0%)	0 (0.0%)	1
Absent	21 (100.0%)	12 (100.0%)	
**SMAD4 Mutation**			
Present	2 (9.5%)	2 (16.7%)	1
Absent	19 (90.5%)	10 (83.3%)	
**SMAD5 Mutation**			
Present	0 (0.0%)	0 (0.0%)	1
Absent	21 (100.0%)	12 (100.0%)	
**SMAD6 Mutation**			
Present	0 (0.0%)	0 (0.0%)	1
Absent	21 (100.0%)	12 (100.0%)	
**SMAD7 Mutation**			
Present	0 (0.0%)	0 (0.0%)	1
Absent	21 (100.0%)	12 (100.0%)	
**SMAD8 Mutation**			
Present	0 (0.0%)	0 (0.0%)	1
Absent	21 (100.0%)	12 (100.0%)	
**SMAD9 Mutation**			
Present	0 (0.0%)	0 (0.0%)	1
Absent	21 (100.0%)	12 (100.0%)	
**BMPR2 Mutation**			
Present	0 (0.0%)	0 (0.0%)	1
Absent	21 (100.0%)	12 (100.0%)	
**BMPER Mutation**			
Present	0 (0.0%)	0 (0.0%)	1
Absent	21 (100.0%)	12 (100.0%)	
**BMP3 Mutation**			
Present	0 (0.0%)	0 (0.0%)	1
Absent	21 (100.0%)	12 (100.0%)	
**BMP2K Mutation**			
Present	0 (0.0%)	0 (0.0%)	1
Absent	21 (100.0%)	12 (100.0%)	
**BMP1 Mutation**			
Present	0 (0.0%)	0 (0.0%)	1
Absent	21 (100.0%)	12 (100.0%)	
**BMP5 Mutation**			
Present	0 (0.0%)	0 (0.0%)	1
Absent	21 (100.0%)	12 (100.0%)	
**BMP10 Mutation**			
Present	0 (0.0%)	0 (0.0%)	1
Absent	21 (100.0%)	12 (100.0%)	
**BMPR1A Mutation**			
Present	0 (0.0%)	0 (0.0%)	1
Absent	21 (100.0%)	12 (100.0%)	
**BMP15 Mutation**			
Present	0 (0.0%)	0 (0.0%)	1
Absent	21 (100.0%)	12 (100.0%)	
**BMP7 Mutation**			
Present	0 (0.0%)	0 (0.0%)	1
Absent	21 (100.0%)	12 (100.0%)	
**BMP23 Mutation**			
Present	0 (0.0%)	0 (0.0%)	1
Absent	21 (100.0%)	12 (100.0%)	
**BMP4 Mutation**			
Present	0 (0.0%)	0 (0.0%)	1
Absent	21 (100.0%)	12 (100.0%)	
**BMP6 Mutation**			
Present	0 (0.0%)	0 (0.0%)	1
Absent	21 (100.0%)	12 (100.0%)	
**BMPR1B Mutation**			
Present	1 (4.8%)	0 (0.0%)	1
Absent	20 (95.2%)	12 (100.0%)	
**BMP8B Mutation**			
Present	0 (0.0%)	0 (0.0%)	1
Absent	21 (100.0%)	12 (100.0%)	
**BMP8A Mutation**			
Present	0 (0.0%)	0 (0.0%)	1
Absent	21 (100.0%)	12 (100.0%)	

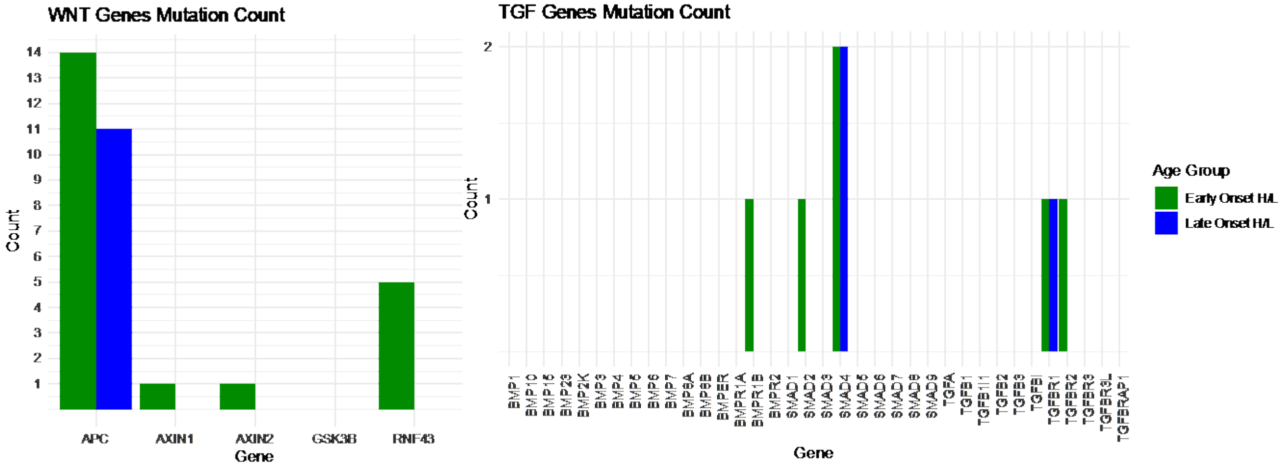

Figure S2. Counts of WNT and TGF-Beta Pathway-Related Gene Alterations in Early-Onset and Late-Onset Hispanic/Latino CRC Patients.

Table S2. Rates of WNT and TGF-Beta pathway alterations in early-onset and late-onset Hispanic/Latino CRC patients, stratified by colon and rectal adenocarcinomas.

Colon Adenocarcinoma	Early Onset n (%)	Late Onset n (%)	p-value
WNT Alterations Present	14 (93.3%)	9 (90.0%)	1
WNT Alterations Absent	1 (6.7%)	1 (10.0%)	
TGF Alterations Present	6 (40.0%)	4 (40.0%)	1
TGF Alterations Absent	9 (60.0%)	6 (60.0%)	

Rectum Adenocarcinoma	Early Onset n (%)	Late Onset n (%)	p-value
WNT Alterations Present	5 (83.3%)	2 (100.0%)	1
WNT Alterations Absent	1 (16.7%)	0 (0.0%)	
TGF Alterations Present	2 (33.3%)	0 (0.0%)	1
TGF Alterations Absent	4 (66.7%)	2 (100.0%)	

Table S3. Alteration Rates of WNT and TGF-Beta Pathway-Related Genes in Early-Onset Hispanic/Latino and Non-Hispanic White CRC Patients.

Gene	Early Onset H/L n (%)	Early Onset NHW n (%)	p-value
**APC Mutation**			
Present	14 (66.7%)	36 (55.4%)	0.5114
Absent	7 (33.3%)	29 (44.6%)	
**AXIN1 Mutation**			
Present	1 (4.8%)	3 (4.6%)	1
Absent	20 (95.2%)	62 (95.4%)	
**AXIN2 Mutation**			
Present	1 (4.8%)	2 (3.1%)	1
Absent	20 (95.2%)	63 (96.9%)	
**GSK3B Mutation**			
Present	0 (0.0%)	0 (0.0%)	1
Absent	21 (100.0%)	65 (100.0%)	
**RNF43 Mutation**			
Present	5 (23.8%)	5 (7.7%)	0.1071
Absent	16 (76.2%)	60 (92.3%)	
**TGFBR2 Mutation**			
Present	1 (4.8%)	1 (1.5%)	0.4309
Absent	20 (95.2%)	64 (98.5%)	
**TGFB2 Mutation**			
Present	0 (0.0%)	2 (3.1%)	1
Absent	21 (100.0%)	63 (96.9%)	
**TGFBRAP1 Mutation**			
Present	0 (0.0%)	2 (3.1%)	1
Absent	21 (100.0%)	63 (96.9%)	
**TGFBR1 Mutation**			
Present	1 (4.8%)	2 (3.1%)	1
Absent	20 (95.2%)	63 (96.9%)	
**TGFBR3 Mutation**			
Present	0 (0.0%)	0 (0.0%)	1
Absent	21 (100.0%)	65 (100.0%)	
**TGFB1I1 Mutation**			
Present	0 (0.0%)	1 (1.5%)	1
Absent	21 (100.0%)	64 (98.5%)	
**TGFBI Mutation**			
Present	0 (0.0%)	0 (0.0%)	1
Absent	21 (100.0%)	65 (100.0%)	
**TGFB1 Mutation**			
Present	0 (0.0%)	0 (0.0%)	1
Absent	21 (100.0%)	65 (100.0%)	
**TGFBR3L Mutation**			
Present	0 (0.0%)	0 (0.0%)	1
Absent	21 (100.0%)	65 (100.0%)	
**TGFB3 Mutation**			
Present	0 (0.0%)	1 (1.5%)	1
Absent	21 (100.0%)	64 (98.5%)	
**TGFA Mutation**			
Present	0 (0.0%)	0 (0.0%)	1
Absent	21 (100.0%)	65 (100.0%)	
**SMAD1 Mutation**			
Present	0 (0.0%)	1 (1.5%)	1
Absent	21 (100.0%)	64 (98.5%)	
**SMAD2 Mutation**			
Present	1 (4.8%)	1 (1.5%)	0.4309
Absent	20 (95.2%)	64 (98.5%)	
**SMAD3 Mutation**			
Present	0 (0.0%)	4 (6.2%)	0.568
Absent	21 (100.0%)	61 (93.8%)	
**SMAD4 Mutation**			
Present	2 (9.5%)	11 (16.9%)	0.5066
Absent	19 (90.5%)	54 (83.1%)	
**SMAD5 Mutation**			
Present	0 (0.0%)	1 (1.5%)	1
Absent	21 (100.0%)	64 (98.5%)	
**SMAD6 Mutation**			
Present	0 (0.0%)	0 (0.0%)	1
Absent	21 (100.0%)	65 (100.0%)	
**SMAD7 Mutation**			
Present	0 (0.0%)	0 (0.0%)	1
Absent	21 (100.0%)	65 (100.0%)	
**SMAD8 Mutation**			
Present	0 (0.0%)	0 (0.0%)	1
Absent	21 (100.0%)	65 (100.0%)	
**SMAD9 Mutation**			
Present	0 (0.0%)	7 (10.8%)	0.1868
Absent	21 (100.0%)	58 (89.2%)	
**BMPR2 Mutation**			
Present	0 (0.0%)	2 (3.1%)	1
Absent	21 (100.0%)	63 (96.9%)	
**BMPER Mutation**			
Present	0 (0.0%)	2 (3.1%)	1
Absent	21 (100.0%)	63 (96.9%)	
**BMP3 Mutation**			
Present	0 (0.0%)	0 (0.0%)	1
Absent	21 (100.0%)	65 (100.0%)	
**BMP2K Mutation**			
Present	0 (0.0%)	0 (0.0%)	1
Absent	21 (100.0%)	65 (100.0%)	
**BMP1 Mutation**			
Present	0 (0.0%)	3 (4.6%)	1
Absent	21 (100.0%)	62 (95.4%)	
**BMP5 Mutation**			
Present	0 (0.0%)	1 (1.5%)	1
Absent	21 (100.0%)	64 (98.5%)	
**BMP10 Mutation**			
Present	0 (0.0%)	1 (1.5%)	1
Absent	21 (100.0%)	64 (98.5%)	
**BMPR1A Mutation**			
Present	0 (0.0%)	1 (1.5%)	1
Absent	21 (100.0%)	64 (98.5%)	
**BMP15 Mutation**			
Present	0 (0.0%)	0 (0.0%)	1
Absent	21 (100.0%)	65 (100.0%)	
**BMP7 Mutation**			
Present	0 (0.0%)	12 (18.5%)	**0.03361**
Absent	21 (100.0%)	53 (81.5%)	
**BMP23 Mutation**			
Present	0 (0.0%)	0 (0.0%)	1
Absent	21 (100.0%)	65 (100.0%)	
**BMP4 Mutation**			
Present	0 (0.0%)	0 (0.0%)	1
Absent	21 (100.0%)	65 (100.0%)	
**BMP6 Mutation**			
Present	0 (0.0%)	0 (0.0%)	1
Absent	21 (100.0%)	65 (100.0%)	
**BMPR1B Mutation**			
Present	1 (4.8%)	0 (0.0%)	1
Absent	20 (95.2%)	65 (100.0%)	
**BMP8B Mutation**			
Present	0 (0.0%)	0 (0.0%)	1
Absent	21 (100.0%)	65 (100.0%)	
**BMP8A Mutation**			
Present	0 (0.0%)	0 (0.0%)	1
Absent	21 (100.0%)	65 (100.0%)	

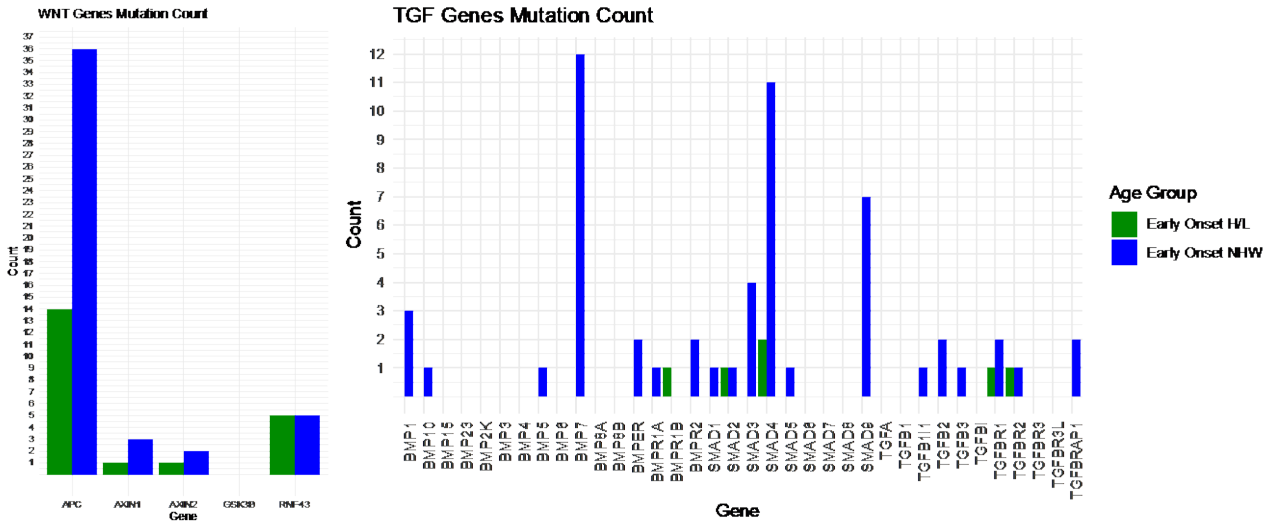

Figure S3. Counts of WNT and TGF-Beta Pathway-Related Gene Alterations in Early-Onset Hispanic/Latino and Non-Hispanic White CRC Patients.

Table S4. Rates of WNT and TGF-Beta pathway alterations in early-onset Hispanic/Latino and Non-Hispanic White (NHW) CRC patients, stratified by colon and rectal adenocarcinomas.

Colon Adenocarcinoma	Early-Onset H/L n (%)	Early-Onset NHW n (%)	p-value
WNT Alterations Present	14 (93.3%)	29 (69.0%)	0.08416
WNT Alterations Absent	1 (6.7%)	13 (31.0%)	
TGF Alterations Present	6 (40.0%)	11 (26.2%)	0.4998
TGF Alterations Absent	9 (60.0%)	31 (73.8%)	

Rectum Adenocarcinoma	Early-Onset H/L n (%)	Early-Onset NHW n (%)	p-value
WNT Alterations Present	5 (83.3%)	15 (65.2%)	0.6328
WNT Alterations Absent	1 (16.7%)	8 (34.8%)	
TGF Alterations Present	2 (33.3%)	6 (26.1%)	1
TGF Alterations Absent	4 (66.7%)	17 (73.9%)	
